# Surprising Effects of Maternal Malaria and Gravidity on Infant Malaria Burden

**DOI:** 10.1371/journal.pmed.0020413

**Published:** 2005-11-08

Every year at least 30 million women in malarious areas of Africa become pregnant. The symptoms and complications of malaria during pregnancy differ with the intensity of malaria transmission and with the level of immunity acquired by the pregnant woman. In areas where malaria is endemic, most adult women have developed sufficient immunity such that, even during pregnancy, P. falciparum infection does not usually result in fever or other clinical symptoms. In these areas, the principal impact of malaria infection is due to the presence of parasites in the placenta. Placental malaria frequently results in low birth weight, and estimates suggest that in endemic areas 19% of cases of infant low birth weight are due to malaria, and that 6% of infant deaths are due to low birth weight caused by malaria.

In addition to affecting birth weight, placental malaria might increase the susceptibility of infants to malaria, but so far studies on this subject have been inconclusive. Patrick Duffy and colleagues examined the effect of placental malaria on infant malaria susceptibility in a prospective cohort study of newborns in a malaria-endemic area. They monitored parasitemia in 453 infants in a region of northeastern Tanzania where malaria transmission is very high (with an estimated 400 infective mosquito bites per individual per year). Sixty-nine of the infants were born to mothers with placental malaria. Placental malaria is caused by a different form of the malaria parasite, which does not commonly infect nonpregnant individuals. Even in endemic areas, women, therefore, lack immunity to the placenta-specific form of the parasite prior to their first pregnancy, but acquire it over successive pregnancies. As a consequence, placental malaria is most frequent and severe in first-time mothers. Of the 69 mothers with placental malaria in this study, 45% were first-time mothers (or primigravid), 38% were giving birth to their second child (secundigravid), and 17% had had multiple prior pregnancies (multigravid).

Overall, infants of mothers with placental malaria were 41% more likely to experience malaria parasitemia themselves in the first year of life. However, the odds of parasitemia throughout infancy were also strongly influenced by the mother's gravidity. The researchers found a surprising protective effect of placental malaria of primigravid mothers on their firstborns' risk of parasitemia. Placental malaria of multigravid women, on the other hand, significantly increased risk of parasitemia during infancy. And even in the absence of placental malaria, firstborn children were less likely to have parasitemia than infants born to multigravid mothers. These results suggest that risk of parasitemia during infancy was modified by an interaction between placental malaria and gravidity. [Fig pmed-0020413-g001]

**Figure pmed-0020413-g001:**
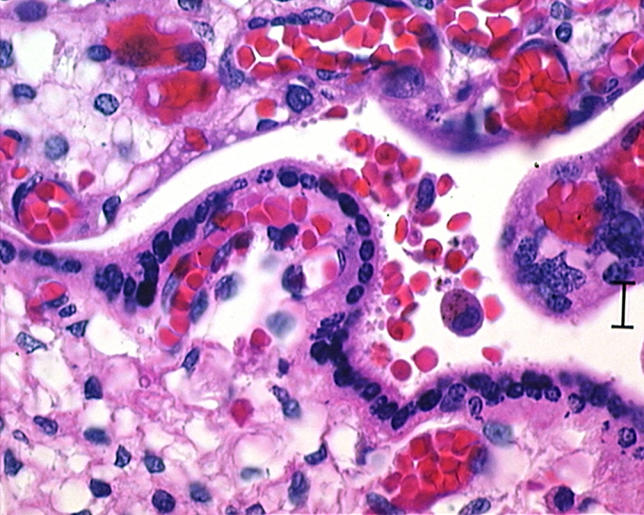
Section from a malaria-infected placenta (Photo: Michal Fried)

Duffy and colleagues speculate that the stronger inflammatory response to placental malaria in first-time mothers could reduce congenital transmission or somehow strengthen the fetal immune system against malaria. They also suggest that the opposing effects of placental malaria in different gravid groups might explain why earlier studies found no significant risk between placental malaria and malaria susceptibility during the first two years of life. The results here are provocative but preliminary. Additional larger studies are necessary to conclusively demonstrate an interaction between placental malaria and gravidity in infant malaria susceptibility, and to examine potential modulation of congenital malaria transmission and infant immunity to the parasite by placental inflammation.

